# Supplementary material for: Chromothripsis during telomere crisis is independent of NHEJ, and consistent with a replicative origin
Source: Genome Res. 2019 May;29(5):737–49. doi: 10.1101/gr.240705.118 (PMC6499312; doi:10.1101/gr.240705.118)
Supplement: Supplemental Material [file supp_gr.240705.118_Supplemental_file_1.zip › contigs/annotated_contigs/DB116/contig.2.DB116_length_547_mean_cov_16.9323583181.docx]

**DB116_length_547_mean_cov_16.9323583181**

```
ACTATTATTTATCAACTATTTATTGTCTTTCCACTAAATAAGAAGTGATCGTCATCTTTTCAGATGAGCACCACAGACTTTGCAAAAGA
 >chr12:9474543-9474801 - E=9e-127
TAATTGCCAATTAACCTTTGTGCCTGGATTTTCTCATTCCTAAAAATAGATAGTATTACATGACTCCAAAGTTTGTTGTGAAGTTAATT

GGGGTGGTGTATCCTAAAGTAACAAAAATGTTGACCACACAGGAGGGGGTGGTTAGTGTAAGACTCTCTGTTTTTCTGT|ACTACT|CA
                                                                                      >
GCTACTCAGGAGGCGAGGCAGAAGAATTGCTCAAACCCCAGAGGTGGAGGTTGCAGTGAGCCAAGATTGTGCCACTGCACTCCAGCCTG
chr12:9473412-9473697 - E=4e-155
GGAAACAGAGCAAGACTCAGTTGAAAAAAAAAGAAACAGAAGAAATTAGCAATATATTTCATTTACTCCAATGTATCCAAATTATGGTC

ATTTTAGCATGTAATCAATATAGATTTTAATGAGATAGTCTATGTTCTTTTTTCCTAAGTCTGAAATTCGGTGTGTATTTACACTGACA

GCACATCTCAATTTA
```
